# Family and Friend Perceptions of Quality of End-of-Life Care in Medicare Advantage vs Traditional Medicare

**DOI:** 10.1001/jamanetworkopen.2020.20345

**Published:** 2020-10-13

**Authors:** Claire K. Ankuda, Amy S. Kelley, R. Sean Morrison, Vicki A. Freedman, Joan M. Teno

**Affiliations:** 1 Department of Geriatrics and Palliative Medicine, Icahn School of Medicine at Mount Sinai, New York, New York; 2 Michigan Center on the Demography of Aging, Institute for Social Research, University of Michigan, Ann Arbor; 3 Division of General Internal Medicine and Geriatrics, School of Medicine, Oregon Health and Science University, Portland

## Abstract

**Importance:**

Medicare Advantage (MA) insures an increasing proportion of Medicare beneficiaries, but evidence is lacking on patient or family perceptions of the quality of end-of-life care in MA vs traditional Medicare.

**Objective:**

To determine if there is a difference in quality of care reported by family and friends of individuals who died while insured by MA vs traditional Medicare at the end of life.

**Design, Setting, and Participants:**

This cross-sectional study used the 2011 to 2017 Medicare-linked National Health and Aging Trends Study to conduct population-based survey research representing 8 668 829 Medicare enrollees. Included individuals were 2119 enrollees who died when aged 65 years or older, with quality of care reported by a family member or close friend familiar with the individual’s last month of life. Analysis was conducted in July 2020.

**Exposures:**

MA enrollment at the time of death or before hospice enrollment.

**Main Outcomes and Measures:**

Perception of end-of-life care was measured with 9 validated items, with the primary outcome variable being overall care rated not excellent. We conducted a propensity score–weighted multivariable model to examine the association of each item with MA vs traditional Medicare enrollment. The propensity score and multivariable model included covariates capturing demographic and socioeconomic factors, function and health, and relationship of the respondent to the individual who died. The sample was then stratified by hospice enrollment and setting of care in the last month.

**Results:**

Of 2119 people in the sample, 670 individuals were enrolled in MA at the time of death or prior to hospice (32.7%) and 1449 were enrolled in traditional Medicare (67.3%). In survey-weighted percentages, 53.6% (95% CI, 51.0% to 56.1%) were women and 43.4% (95% CI, 41.5% to 45.3%) were older than 85 years at the time of death. In the adjusted model, family and friends of individuals in MA were more likely to report that care was not excellent (odds ratio, 1.28; 95% CI, 1.01 to 1.61; *P* = .04) and that they were not kept informed (odds ratio, 1.48; 95% CI, 1.06 to 2.05; *P* = .02). For those in nursing homes, there was an estimated probability of 57.2% of respondents reporting that care was not excellent for individuals with traditional Medicare, compared with 77.9% of respondents for individuals with MA (marginal increase for those in MA, 0.21; 95% CI, 0.08 to 0.32; *P* = .001).

**Conclusions and Relevance:**

In this cross-sectional study of people who died while enrolled in Medicare, friends and family of those in MA reported lower-quality end-of-life care compared with friends and family of those enrolled in traditional Medicare. These findings suggest that, given the rapid growth of MA, Medicare should take steps to ensure that MA plans are held accountable for quality of care at the end of life.

## Key Points

**Question:**

Does the quality of care at the end of life as reported by bereaved family and friends differ for people enrolled in Medicare Advantage vs traditional Medicare at the end of life?

**Findings:**

In this cross-sectional study of 2119 individuals who died while enrolled in Medicare, the family and friends of people who were enrolled in Medicare Advantage were more likely to report that care was not excellent and that they were not kept informed in the last month of life compared with family and friends of those enrolled in traditional Medicare.

**Meaning:**

In this study, family and friends of people who died while enrolled in Medicare Advantage reported lower quality of care in the last month of life compared with family and friends of those who died while enrolled in traditional Medicare.

## Teaser

This cross-sectional study examines friend and family perceptions of quality of end-of-life care for individuals enrolled in Medicare Advantage at time of death vs those enrolled in traditional Medicare.

## Introduction

The Medicare Advantage (MA) program is growing rapidly, yet its association with the quality of care for older adults with serious illness is unknown. From 2008 to 2018, the proportion of Medicare beneficiaries enrolled in MA plans increased from 21% to 34%.^1^ MA plans must cover the same services as traditional Medicare but often offer lower cost-sharing rates and an expanded array of benefits, such as vision, hearing, and dental care.^2^ MA plans can offer these enhanced benefits because the program can better control costs, by limiting networks, using prior-authorization procedures, and actively managing services provided to beneficiaries.^3^

Despite the lower cost-sharing rates and the expanded benefits that MA plans may offer, there is evidence that individuals with serious illnesses may experience lower-quality care in MA plans. Older adults with MA are more likely to be discharged from acute care settings to lower-quality skilled nursing facilities and use lower-quality home health agencies compared with older adults with traditional Medicare.^4,5^ Additional studies have demonstrated that as select populations of older adults become ill, they are more likely to leave MA plans; these populations include older adults with new cancer diagnoses, kidney failure treated with dialysis, new functional disability, and high costs of care.^6,7,8,9^ Patients may switch plans like this because of barriers to care through MA.

Our study of the quality of end-of-life care in MA plans comes as the Centers for Medicare & Medicaid Services is set to start testing a “carve-in” of hospice services in 2021, meaning that hospice will be a covered benefit within MA and therefore MA plans will take a more active role in hospice services.^10^ Currently, the hospice benefit is “carved out” from MA, although MA plans are still involved in the care of hospice enrollees through several mechanisms. Older adults in MA are more likely to receive hospice services, potentially through direct care coordination or by contracting with clinicians or facilities more likely than MA to refer to hospice.^11,12^ The MA plan is permitted to guide beneficiaries to specific hospices and oversee aspects of their care, but it is not permitted to restrict access to a defined hospice network. When an MA beneficiary elects hospice, traditional Medicare will then cover most benefits, including hospice, but the MA plan will still cover drugs and treatments for conditions unrelated to the hospice terminal diagnosis, as well as any supplementary benefits and cost sharing that are part of the MA plan.

Given the lack of data on quality of end-of-life care in MA and the increased role of MA plans in hospice, we aimed to assess whether quality of care as reported by family and friends in the last month of life is different for individuals insured by MA vs those insured by traditional Medicare at the end of life. Understanding the care experienced overall by those in MA across settings, from the individual’s home to nursing homes to hospice, may help assess the implication of the potential policy change of including hospice in MA. We therefore aimed to assess whether rates of family and friend perception of quality of care in the last month of life differed for the family or friends of those in MA vs those in traditional Medicare plans.

## Methods

This cross-sectional study used the National Health and Aging Trends Study (NHATS), which uses a mortality follow-back survey and directly queries proxies of individuals who died about the person’s experience in the last month of life. The Johns Hopkins University institutional review board approved the NHATS protocol, and all participants provided written informed consent. The Icahn School of Medicine at Mount Sinai institutional review board and the Centers for Medicare & Medicaid Services privacy board approved this cross-sectional study, which followed the Strengthening the Reporting of Observational Studies in Epidemiology (STROBE) reporting guideline for cross-sectional studies.

The NHATS is an annual, longitudinal survey of a nationally representative cohort of adults in the United States aged 65 years and older that began in 2011. This survey closely tracks mortality among respondents, and if a respondent dies, a close family member or friend is interviewed as a proxy about the care the individual received in the last month of life. The response rates for NHATS interviews covering the last month of life ranged from 93.8% (in 2012) to 97.1% (in 2015).^13^

We included individuals in the survey who died and whose friends or family conducted an interview covering the last month of life from 2011 to 2017. From the 2810 individuals who died during this period, we excluded 267 individuals for whom proxies reported that they were a little or not at all familiar with the individual’s last month of life. We excluded 424 observations from individuals for whom the proxy reporter was not a friend or family member, such as employees of a nursing home or nonfamilial legal guardians. We used NHATS-linked Medicare files to determine enrollment in MA vs traditional Medicare in the last month of life. If an individual died in hospice, we considered the person to have MA if enrolled in MA before hospice enrollment. We did this because the MA plan played a major role in the care plan before hospice, guided the patient to a specific hospice, and may have overseen aspects of care in hospice. Finally, owing to evidence of individuals with higher need switching from MA to traditional Medicare near end of life, we tested an expanded definition of MA that considered insurance status 12 months before death rather than simply at time of death.

### Measures of Quality of Care in the Last Month of Life

Quality of end-of-life care as reported by friends and family was assessed in the NHATS Last Month of Life interview with validated items from a National Quality Forum measure that address the following domains: pain and symptom management, communication and decision-making, emotional support, and an overall quality rating.^14^ For pain- and symptom-management questions, proxies were asked whether the individual who died experienced the symptom in the last month of life. If so, the proxy was asked whether the individual received the right amount of help for the symptom. Proxies were additionally asked whether the individual was treated with respect, whether any decisions were made that the individual would not have wanted (we described this as *care not consistent with goals*), whether decisions were made without the individual’s or family’s input (we described this as *inadequate communication*), and how often family members were kept informed about the individual’s condition.

We defined low-quality care as the proxy reporting any of the following: the individual did not receive the right amount of help for symptoms; the individual was not always treated with respect; decisions were made that the individual would not have wanted; decisions were made without the individual’s or family’s input; or family members were not always kept informed about the individual’s condition. Proxies were asked to rate the overall quality of care in the last month of life as excellent, very good, good, fair, or poor. The primary study outcome was a report that overall care in the last month of life was not excellent. This measure has been used as a summary marker of the quality of end-of-life care in hospice as reported by proxies.^14^ These quality measures in the NHATS Last Month of Life interview have been used in studies assessing trends in quality, racial disparities, late-life transitions, and goal-concordant care.^15,16,17,18^

We additionally collected information on patterns of care at the end of life. Measures collected included enrollment in hospice, live discharge from hospice, and length of stay in hospice, all determined from the Medicare hospice claims. We determined that an individual who died made a late-life transition if the proxy reported that the individual was in the setting of care where death occurred for 3 days or less.^17^ Proxies were asked what the location of death was (ie, home, hospice facility, hospital or in transit to hospital, or nursing home), how long the individual was at this last location, and where the individual was before moving to the location where death occurred. This information allowed us to identify individuals who died at home or in a hospital vs another location, as well as those who were in a nursing home or hospital either at the time of death or before death but within the last month of life.

### Covariates

Covariates collected during the NHATS Last Month of Life interview included age at death, sex, race/ethnicity (as determined by self-report in NHATS survey), bedbound status in the last month of life (defined as getting out of bed some days, rarely, or not at all), location of death (ie, home or hospice facility, hospital or in transit to hospital, or nursing home), and relationship of proxy to the individual (ie, spouse, child, or friend or other family member). Additional covariates were collected in the NHATS round from before the individual’s death, with measures including income, residence in a metropolitan region, receiving help for any activities of daily living (ie, eating, bathing, using the toilet, transferring into and out of bed, or dressing), and self-reported medical conditions of lung disease, stroke, or cancer. Probable dementia was determined from self-report and cognitive testing through a validated measure assessed during the last NHATS interview before the individual’s death.^19^ Medicaid status at the time of death was determined from linked Medicare files.

### Statistical Analysis

To describe individuals enrolled in MA at the end of life, we first compared the demographic characteristics, regional characteristics, and health and function of individuals who died while enrolled in MA vs traditional Medicare. We then developed a propensity score using the aforementioned covariates to estimate enrollment in MA at the end of life. The selected covariates captured the known differences across the populations of people who died and were enrolled in MA or traditional Medicare, with those in MA more likely to be younger, members of racial/ethnic minority groups, and low income and less likely to be functionally disabled.^20^

We additionally included the survey weight as a covariate in the propensity score to account for the complex survey design and to ensure that our results were generalizable to the Medicare population.^21^ The propensity score achieved good balance across the MA and traditional Medicare groups; however, we found imbalance in the covariates for lung disease, stroke, and cancer across the blocks of the propensity score. Given that there was a statistically significant difference in rates of lung disease between populations, a propensity score was tested that included only this comorbidity. However, it still did not achieve balance across all blocks of the propensity score. Given the low theoretical importance of these covariates, they were not included in the model.^22^

From the propensity score model, we then derived the inverse probability of treatment weights, normalized these weights, and multiplied them by the survey weights.^21^ For each variable for quality of end-of-life care, we conducted a separate logistic regression, weighting by the combined survey and inverse propensity of treatment weight, with all covariates as well as enrollment in MA as estimators (eAppendix in Supplement). We separately conducted an unadjusted (only survey-weighted) comparison, a logistic regression model (using all covariates in the propensity score model), and a propensity-weighted regression model to compare these approaches.

Because this was a sensitivity analysis and the individuals with the most severe illnesses might leave MA plans at the very end of life,^6^ we determined MA status from 1 year before death. Given that hospice is carved out from MA,^10^ we repeated the analysis for the outcome of care rated not excellent at the end of life, stratifying by hospice enrollment. We additionally stratified by setting of death: dying at home (vs not at home), care in a nursing home at death or within the last month of life, and care in a hospital at death or within the last month of life. Analysis was conducted using Stata statistical software version 16.0 (StataCorp) with complex survey commands to account for the survey design and sampling approach.^23,24^ For all analyses, survey-weighted statistics are reported unless otherwise specified. Two-sided logistic regression was used to evaluate *P* values, with a significance threshold of *P* < .05. Analysis was conducted in July 2020.

## Results

We identified 2119 individuals in NHATS who died while enrolled in Medicare between 2011 and 2017, with 670 of those individuals (weighted proportion, 32.7%) enrolled in MA at the time of death or before hospice enrollment (Table 1). In survey-weighted percentages for the study population, 53.6% (95% CI, 51.0% to 56.1%) were women, 43.4% (95% CI, 41.5% to 45.3%) were older than 85 years at the time of death, and 80.1% (95% CI, 77.4% to 82.5%) had reported that they were White and not Hispanic. This population was poorer than the overall Medicare population, with 40.3% (95% CI, 37.1% to 43.5%) in the lowest income quartile for all Medicare beneficiaries. Of 2119 patients, 83.9% (95% CI, 81.7% to 85.9%) had impaired activities of daily living in the last NHATS survey conducted before death and 1.0% (95% CI, 38.6% to 43.4%) were bedbound in the last month of life.

**Table 1.** Patient Characteristics^a^

Characteristic	% (95% CI)			*P* value^b^
	Total (N =2119)	Traditional Medicare (n = 1449)	MA (n = 670)	
National estimate, No. (%)	8 668 829	5 832 889 (67.3)	2 835 940 (32.7)	
Age, y				
65-74	21.1 (19.0-23.4)	20.0 (17.5-22.8)	23.4 (18.9-28.6)	.11
75-85	35.5 (33.5-37.5)	34.5 (32.1-36.9)	37.7 (32.7-42.9)	
>85	43.4 (41.5-45.3)	45.6 (43.2-47.9)	39.0 (35.3-42.8)	
Women	53.6 (51.0-56.1)	53.5 (50.6-56.5)	53.6 (48.5-58.6)	.98
Race/ethnicity				
White, non-Hispanic	80.1 (77.4-82.5)	82.4 (79.5-85.0)	75.3 (70.1-79.8)	.01
Black, non-Hispanic	9.9 (8.8-11.2)	9.7 (8.2-11.4)	10.5 (8.7-12.6)	
Other race/ethnicity, non-Hispanic	3.6 (2.5-5.2)	2.7 (1.6-4.5)	5.4 (3.3-8.7)	
Hispanic	6.4 (5.0-8.1)	5.2 (3.8-7.1)	8.8 (6.1-12.4)	
Quartile of income^c^				
Lowest	40.3 (37.1-43.5)	37.2 (33.5-41.1)	46.4 (41.1-51.7)	.03
Second	28.8 (25.9-31.9)	29.1 (25.9-32.5)	28.2 (24.2-32.7)	
Third	20.0 (18.4-21.7)	21.0 (18.8-23.3)	18.0 (13.6-23.6)	
Highest	10.9 (9.0-13.3)	12.8 (9.8-16.4)	7.4 (5.0-10.8)	
Enrolled in Medicaid at death	21.0 (18.6-23.8)	20.8 (18.0-23.9)	21.6 (17.4-26.5)	.76
Enrolled in an MA special needs plan^d^	1.9 (1.1-3.1)	NA	5.8 (3.5-9.4)	NA
Residence in a metropolitan region	81.8 (73.2-88.0)	78.3 (68.3-85.9)	88.9 (81.8-93.5)	.002
Health and function				
Bedbound in last month of life^e^	41.0 (38.6-43.4)	41.7 (38.4-45.1)	39.4 (34.5-44.5)	.50
Impaired activity of daily living^f^	83.9 (81.7-85.9)	85.1 (82.6-87.2)	81.5 (76.6-85.5)	.15
Lung disease^g^	8.3 (6.9-9.9)	7.1 (5.6-8.9)	10.8 (8.4-13.7)	.01
Stroke^g^	8.9 (7.6-10.3)	9.5 (8.1-11.1)	7.5 (5.6-10.0)	.12
Cancer^g^	18.8 (16.7-21.0)	19.2 (16.8-21.9)	17.9 (14.3-22.1)	.55
Probable dementia on prior survey^h^	37.8 (35.0-40.7)	40.3 (36.4-44.2)	32.9 (29.7-36.3)	.004

Abbreviation: MA, Medicare Advantage; NA, not applicable.

^a^ For individuals enrolled in hospice at the time of death, MA status was ascertained from the month prior to hospice enrollment.

^b^ *P* values determined through χ^2^ tests, adjusting for survey design and sampling approach.

^c^ Quartile of income is defined for the entire Medicare population.

^d^ MA special needs plans are specific plans for individuals with chronic or disabling conditions (14 individuals in the sample), dual-eligibles (48 individuals), and those residing in institutions (20 individuals).

^e^ Defined as getting out of bed some days, rarely, or not at all in the last month of life.

^f^ Self-reported by last National Health and Aging Trends Study survey before death and defined as receiving help with eating, bathing, using the toilet, transferring into and out of bed, or dressing.

^g^ Self-reported on the last National Health and Aging Trends Study survey before death.

^h^ Determined by self-report and cognitive testing on the last National Health and Aging Trends Study survey before death.

Individuals with MA, compared with those with traditional Medicare, were less likely to be non-Hispanic White (75.3% [95% CI, 70.1% to 79.8%] vs 82.4% [95% CI, 79.5% to 85.0%]; *P* = .01) and have dementia (32.9% [95% CI, 29.7% to 36.3%] vs 40.3% [95% CI, 36.4% to 44.2%]; *P* = .004) and more likely to report the lowest quartile of income on the NHATS round before death (46.4% [95% CI, 41.1% to 51.7%] vs 37.2% [95% CI, 33.5% to 41.1%]; *P* = .03), reside in a metropolitan region (88.9% [95% CI, 81.8% to 93.5%] vs 78.3% [95% CI, 68.3% to 85.9%]; *P* = .002), and have lung disease (10.8% [95% CI, 8.4% to 13.7%] vs 7.1% [95% CI, 5.6% to 8.9%]; *P* = .01).

**Figure 1.**
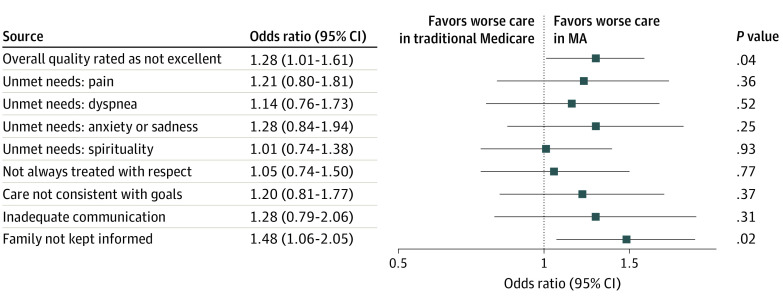
Family- or Friend-Reported Quality of Care in the Last Month of Life Figure displays odds ratios with 95% CIs. Model is weighted by survey weights, which account for survey design and sampling approach, and a propensity score weight, which estimates the odds of Medicare Advantage (MA) enrollment. The propensity score and overall model include covariates for age, sex, race/ethnicity, income quartile, Medicaid enrollment at time of death, residence in a metropolitan region, bedbound status in last month, impaired activities of daily living on last National Health and Aging Trends Study survey, dementia on last National Health and Aging Trends Study survey, and relationship of proxy to the individual who died.

**Figure 2.**
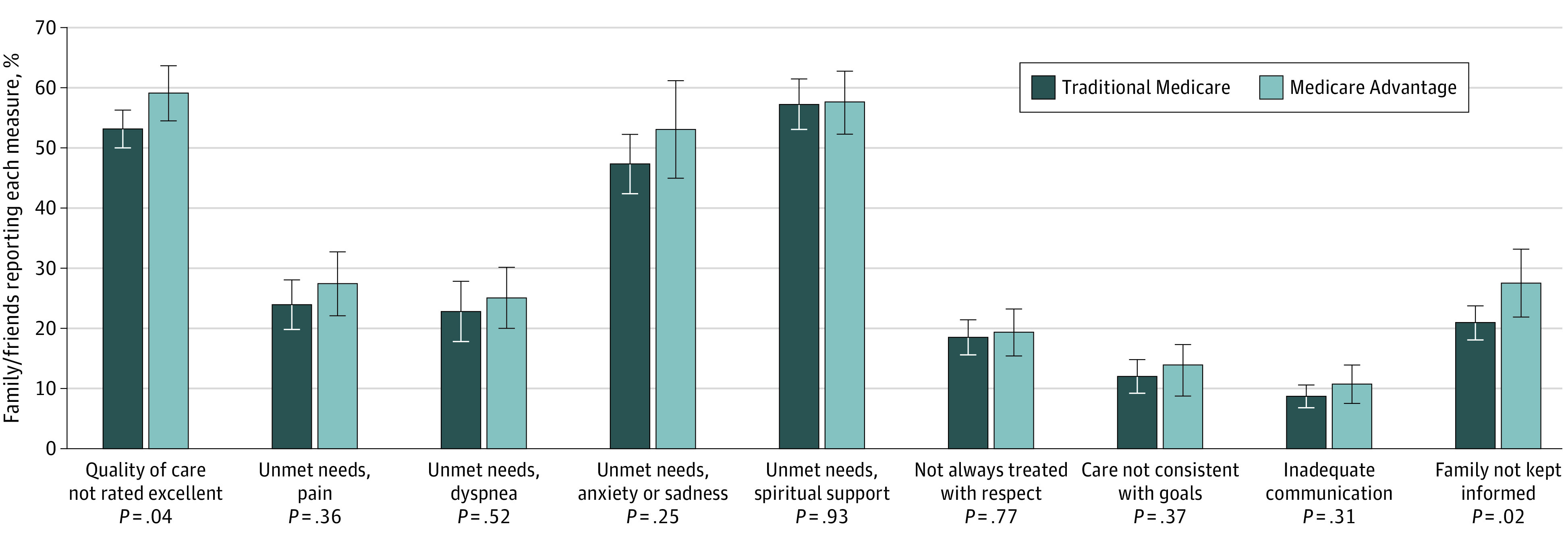
Estimated Proportions Reporting Quality Measures Model is weighted by survey weights, which account for survey design and sampling approach, and a propensity score weight, which estimates the odds of Medicare Advantage enrollment. The propensity score and overall model include covariates for age, sex, race/ethnicity, income quartile, Medicaid enrollment at time of death, residence in a metropolitan region, bedbound status in last month, impaired activities of daily living on last National Health and Aging Trends Study survey, dementia on last National Health and Aging Trends Study survey, and relationship of proxy to the individual who died. Whiskers indicate 95% CIs.

As demonstrated in the propensity score–weighted model (Figure 1), the bereaved family or friends of those who died while in MA were more likely to report that the overall quality of care was not excellent (adjusted odds ratio, 1.28; 95% CI, 1.01 to 1.61; *P* = .04.). This translates to an estimated probability of 53.1% (95% CI, 50.0% to 56.3%) of respondents reporting that care was not excellent for individuals with traditional Medicare, compared with 59.0% (95% CI, 54.4% to 63.5%) for those with MA (Figure 2 and eTable 1 in the Supplement). For individuals with MA, friends and family were also more likely to report that they were not kept informed in the last month of life (adjusted odds ratio, 1.48; 95% CI, 1.06 to 2.05; *P* = .02). Differences in other indicators were not statistically significant.

**Figure 3.**
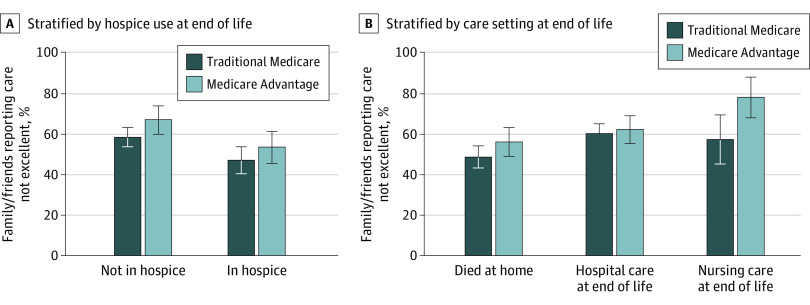
Estimated Proportions Reporting Care Was Not Excellent in the Last Month of Life, Stratified by Subgroups Figure displays estimated proportions with 95% CIs. Model is weighted by survey weights, which account for survey design and sampling approach, and a propensity score weight, which estimates the odds of MA enrollment. The propensity score and overall model include covariates for age, sex, race/ethnicity, income quartile, Medicaid enrollment at time of death, residence in a metropolitan region, bedbound status in last month, impaired activities of daily living on last National Health and Aging Trends Study survey, dementia on last National Health and Aging Trends Study survey, and relationship of proxy to the individual who died.

In stratified analysis for individuals with MA or traditional Medicare, estimated probability of family or friends reporting that care was not excellent was higher for individuals not in hospice compared to those in hospice (marginal increase for those in hospice, −0.13; 95% CI, −0.20 to −0.05; *P* = .001) (Figure 3 and eTable 4 in the Supplement). However, the estimated probability of reporting that care was not excellent was higher for individuals with MA vs traditional Medicare, regardless of hospice enrollment (marginal increase for those in MA, 0.68; 95% CI, 0.17 to 0.12; *P* = .01). Stratified by care settings (ie, died at home vs not at home, hospital care at end of life vs no hospital care at end of life, and nursing home care at end of life vs no nursing home care at end of life), there is a significant gap between MA and traditional Medicare for those who received care in the nursing home at the end of life: there was an estimated probability of 57.2% that family or friend respondents of individuals with traditional Medicare would report that care was not excellent, compared with 77.9% for family or friend respondents of those with MA (marginal increase for those in MA, 0.21; 95% CI, 0.08 to 0.32; *P* = .001). The estimated rates of each measure of quality of end-of-life care were similar across survey-weighted, survey-weighted regression, and survey and propensity–weighted regression models (eTable 3 in the Supplement).

There were no differences in rates of hospice use, rates of short stays (≤3 days) in hospice, live discharges from hospice, or late-life transitions in care for individuals with MA vs traditional Medicare (Table 2). Individuals with MA, compared with those with traditional Medicare, were more likely to die at home or in a hospice unit (51.6% [95% CI, 47.1% to 56.2%] vs 46.3% [95% CI, 43.5% to 49.2%]) and less likely to die in a nursing home (12.3% [95% CI, 10.0% to 15.1%] vs 20.5% [95% CI, 18.1% to 23.1%]) (*P* < .001). Individuals who died while in MA, compared with those with traditional Medicare, were more likely to receive hospital care at the end of life (42.0% [95% CI, 37.3% to 47.0%] vs 36.3% [95% CI, 33.7% to 39.0%]; *P* = .04) and less likely to receive nursing home care at the end of life (14.0% [95% CI, 11.3% to 17.3%] vs 23.8% [95% CI, 21.4% to 26.3%]; *P* < .01). There were no differences when MA enrollment was defined as MA status at 12 months before death (eTable 2 in the Supplement).

**Table 2.** Differences in End-of-Life Care

	% (95% CI)		*P* value
	Rate in traditional Medicare	Rate in MA	
Total	67.3 (63.0-71.3)	32.7 (28.7-37.0)	NA
Any hospice use	49.8 (46.8-52.7)	54.2 (48.5-59.7)	.16
Stay of ≤3 d	24.1 (20.5-28.1)	26.6 (21.4-32.5)	.42
Stay of ≤30 d	65.4 (60.6-69.9)	66.3 (60.5-71.6)	.82
Live discharge	44.4 (40.2-48.7)	46.1 (39.7-52.7)	.66
Late-life transition^a^	16.8 (14.6-19.2)	17.8 (14.2-22.0)	.63
Residence at time of death			
Home or hospice unit	46.3 (43.5-49.2)	51.6 (47.1-56.2)	<.001
Hospital	33.2 (30.6-35.8)	36.1 (31.8-40.6)	
Nursing home	20.5 (18.1-23.1)	12.3 (10.0-15.1)	
Any hospital care at end of life	36.3 (33.7-39.0)	42.0 (37.3-47.0)	.04
Any nursing home care at end of life	23.8 (21.4-26.3)	14.0 (11.3-17.3)	<.001
Quality of care not rated excellent	52.9 (49.8-56.0)	59.1 (54.3-63.7)	.03

Abbreviations: MA, Medicare Advantage; NA, not applicable.

^a^ A change in location within the last 3 days of life. Either place of death was a hospital or the individual was in hospital immediately before transitioning to the place of death and within the last 30 days of life. Either place of death was a nursing home or the individual was in a nursing home immediately before transitioning to the place of death and within the last 30 days of life.

## Discussion

The expansion of the MA program is demonstrative of federal efforts in the United States to shift to value-based payment mechanisms that increase flexibility and attempt to control costs while rewarding high-quality care. However, despite 15 years of increasing use of MA, little is known about the quality of care for people who are seriously ill or dying in MA vs in traditional Medicare. Using NHATS interviews with family or close friends of people who died, this cross-sectional study found that the family and friends of individuals insured by MA plans perceived lower-quality care at the end of life compared with their counterparts with traditional Medicare, regardless of participation in hospice. We found multiple indicators of lower perceived quality of end-of-life care in MA, particularly for those who received care in nursing homes in the last month of life. These results raise concerns about the experience of this population, which has severe health issues, and attest to the importance of quality measures that capture patient perceptions of quality of care.

There are several reasons why perceived quality of care at the end of life may be worse for individuals enrolled in MA. Prior studies have demonstrated the strong influence MA plans have on postacute and institutional care, such as skilled nursing, home health, and nursing home care.^3,4,5^ MA plans may be restricting their networks to facilities and agencies that are willing to accept lower prices and that consequently may cut staff or other expenses important to the perceived quality of care of these older adults, who are at increased risk. This hypothesis is supported by evidence^4,5^ that MA plans refer to skilled nursing facilities and home health agencies with lower ratings for quality. It is additionally supported by our finding of a significant gap in perceived quality between MA and traditional Medicare for individuals residing in nursing homes at the end of life. While we did find that perceived quality of care was greater for individuals enrolled in hospice, MA enrollees in hospice still experienced lower perceived quality of care than traditional Medicare enrollees in hospice. This may be because most hospice stays are less than 2 weeks or because MA plans still steer beneficiaries to specific hospices and may oversee elements of care in hospice.

These findings arrive ahead of the planned Medicare demonstration of a carve-in of hospice to the MA program, as part of the Value-Based Insurance Design Model. As part of the demonstration, MA plans will be held accountable for the quality of end-of-life care, which presents an opportunity for quality improvement. Our results suggest that it is critical that Medicare conduct mortality follow-back surveys that directly capture the experience of the bereaved. Through appropriate quality measurement, reporting, and payment incentives, MA plans could be incentivized to limit the care delivered by health care facilities (including nursing facilities) and clinicians that provide low-quality care at the end of life and to implement supports to improve quality of care, such as through geriatric and palliative care teams.

### Limitations

This study has some limitations. Given that this is a cross-sectional, observational study, further experimental and quasi-experimental studies are necessary to establish causality. It is possible that differences were driven by unmeasured factors driving enrollees to choose MA plans. We use a propensity score–weighted model to adjust for differences in selection of MA plans based on demographic, socioeconomic, and health characteristics, but it is difficult to fully account for differences between individuals enrolled in MA and those in traditional Medicare given the highly regional variations in enrollment rates of MA and the reduced cost-sharing activity in MA plans. We accounted for end-of-life selection out of MA, which is likely to occur in individuals with the most severe conditions, through a sensitivity analysis that attributes individuals to MA if they are in the program 1 year before death. However, there may still be selection out of MA in the 2 to 3 years before death for individuals with chronic illnesses. Given the evidence that beneficiaries with more severe health issues and higher costs tend to leave MA plans, any unmeasured differences likely underestimate the negative association between MA plans and perceptions of quality.

Because of our sample size and data limitations, we were not able to compare types of MA plans, such as special needs plans vs health maintenance organization or preferred provider organization plans. Similarly, owing to sample size and geographic restrictions on NHATS data, we were unable to account for state-level variations or other regional differences in MA penetration. While we were able to compare quality by setting of care using proxy reports, we were unable to use claims, which are likely more accurate given that MA encounter data are not available for NHATS enrollees. Additionally, this study relies on proxy reports, not patient reports, of the quality of end-of-life care. We included only family and close friends familiar with the individual’s end-of-life care using validated quality measures, which may bias findings. The experience of bereaved family members and close friends at the end of life is a focus of palliative care and efforts to improve care for serious illness, suggesting that family and friend assessments and perceptions should be used in assessing quality of end-of-life care.

### Conclusions

Our findings suggest that patients at the end of life, a population with serious illness and high needs, experience lower quality of care in MA as perceived by family and close friends. This should draw the attention of Medicare, as well as state Medicaid programs, which are highly invested in the care of this population. Medicare and states could systematically assess quality of end-of-life care, including through direct reports from family and close friends, and carefully monitor the quality of hospice care under a potential future hospice carve-in to protect these individuals and their caregivers. While MA plans may increasingly appeal to older adults because of these plans’ lower costs and expanded benefits, MA plans must also offer equal or improved quality of care to those in the last days of life.

## Notes

Supplement. **eAppendix.** Additional Technical Details on Analysis Approach, Including Model Fit **eTable 1.** Predicted Rates of Each Measure of Quality of End-of-life Care, From Propensity Score Weighted Model **eTable 2.** Results of Sensitivity Analysis Defining Medicare Advantage Enrollment at 1 Year Prior to Death **eTable 3.** Comparing Survey Weighted Regression Model and Combined Survey/propensity Weighted Regression Models **eTable 4.** Full Specifications of the Stratified Analyses Conducted by Hospice Status and Location of Death
