# Supplementary material for: Family and Friend Perceptions of Quality of End-of-Life Care in Medicare Advantage vs Traditional Medicare
Source: JAMA Netw Open. 2020 Oct 13;3(10):e2020345. doi: 10.1001/jamanetworkopen.2020.20345 (PMC7877489; doi:10.1001/jamanetworkopen.2020.20345)
Supplement: Supplement. — eAppendix. Additional Technical Details on Analysis Approach, Including Model Fit eTable 1. Predicted Rates of Each Measure of Quality of End-of-life Care, From Propensity Score Weighted Model eTable 2. Results of Sensitivity Analysis Defining Medicare Advantage Enrollment at 1 Year Prior to Death eTable 3. Comparing Survey Weighted Regression Model and Combined Survey/propensity Weighted Regression Models eTable 4. Full Specifications of the Stratified Analyses Conducted by Hospice Status and Location of Death [file jamanetwopen-e2020345-s001.pdf]

# Supplemental Online Content

Ankuda CK, Kelley AS, Morrison RS, Freedman VA, Teno JM. Family and friend perceptions of quality of end-of-life care in Medicare Advantage vs traditional Medicare. *JAMA Netw Open*. 2020;3(10):e2020345. doi:10.1001/jamanetworkopen.2020.20345

**eAppendix.** Additional Technical Details on Analysis Approach, Including Model Fit

**eTable 1.** Predicted Rates of Each Measure of Quality of End-of-life Care, From Propensity Score Weighted Model

**eTable 2.** Results of Sensitivity Analysis Defining Medicare Advantage Enrollment at 1 Year Prior to Death

**eTable 3.** Comparing Survey Weighted Regression Model and Combined Survey/propensity Weighted Regression Models

**eTable 4.** Full Specifications of the Stratified Analyses Conducted by Hospice Status and Location of Death

This supplemental material has been provided by the authors to give readers additional information about their work.

© 2020 Ankuda CK et al. *JAMA Network Open.*

**eAppendix.** Additional Technical Details on Analysis Approach, Including Model Fit

a. <u>Develop a propensity score to predict enrollment in MA at the end of life, including survey weight</u>
As described in the manuscript, we included covariates capturing demographics, socioeconomic status, and health that have been previously demonstrated to vary between the MA and TM decedent populations. This initially included the following variables: age at death (categorized as 65-74. 75-84, and 85+), female, white race, income quartile at survey prior to death, enrolled in Medicaid at the time of death, residing in a metropolitan vs. non-metropolitan region at survey prior to death, difficulty getting out of bed at time of death, receiving help for an activity of daily living (bathing, toileting, transferring, eating, dressing) at survey prior to death, probable dementia at survey prior to death, prior diagnosis of lung disease, prior diagnosis of stroke, prior diagnosis of cancer, if the last month of life survey was conducted with a spouse, child, or other individual, and survey weight (as recommended by DuGoff, et al).

b. <u>Refine propensity score to achieve balance across MA and TM groups</u>
We first assessed the balance of the propensity score over the MA vs. TM groups. In the specification of the model with 5 blocks across the propensity score, the mean propensity score was not different for the treated (individuals in MA) vs. controls (individuals in TM) in each block, with a p-value threshold of 0.05.

We then assessed the balance of covariates within the propensity score over each block of the propensity score. We found there was an imbalance in covariates across blocks for three variables: cancer, lung disease, and stroke. Given that there was a statistical difference in rates of lung disease between the MA and TM populations, a propensity score was tested that included only this comorbidity, but it still did not achieve balance across all blocks of the propensity score. We therefore revised the propensity score to exclude these variables, given there was not a strong theoretical argument to keep them in the model and their low frequency (see Table 1) likely led to the imbalance. The revised propensity score achieved balance across all quintiles except for Block 2, which was split. With this specification, there was balance across all propensity score blocks:

| Group | Obs- Group 0 | Obs- Group 1 | Mean- group 0 | Mean- group 1 | p-value for differences in mean score between groups |
|---|---|---|---|---|---|
| 1 | 140 | 33 | 0.165 | 0.170 | 0.35 |
| 2 | 279 | 86 | 0.252 | 0.259 | 0.05 |
| 3 | 242 | 107 | 0.327 | 0.329 | 0.21 |
| 4 | 198 | 132 | 0.001 | 0.001 | 0.33 |
| 5 | 238 | 196 | 0.450 | 0.450 | 0.94 |

Additionally, there was balance for all covariates in the 5 propensity score blocks, with a threshold of $p<0.05$.

c. <u>Derive a new weight from the inverse probability of treatment weight and survey weight</u>
We first used a double robust estimator for each outcome variable, including the covariates in the propensity score as above. This was then normalized to range from 0 to 1 and multiplied by the survey weight. This resulted in a new weight variable, specific to each of the study outcome variables.

d. <u>Conduct separate logistic regressions for each outcome variable</u>
For each outcome variable, we estimated a logistic regression model that included all the above covariates and enrollment in MA at the time of death. This model was conducted using the Stata survey commands, including the sampling unit and stratum. The weight was the new weight described in part c above. For two models (that predicting unmet spiritual needs and predicting unmet dyspnea needs), there were missing standard errors because of a stratum with a single sampling unit. For these models, the stratum with a single sampling unit was combined with an adjacent sampling unit to allow the model to run.

e. <u>References for this modeling approach:</u>

© 2020 Ankuda CK et al. *JAMA Network Open.*

Garrido MM, Kelley AS, Paris J, et al. Methods for constructing and assessing propensity scores. *Health Serv Res*. 2014;49(5):1701-1720. doi:10.1111/1475-6773.12182

Dugoff EH, Schuler M, Stuart EA. Generalizing observational study results: applying propensity score methods to complex surveys. *Health Serv Res*. 2014;49(1):284-303. doi:10.1111/1475-6773.12090

Statalist: The Stata Forum. "Propensity score analysis for complex survey data using PSCORE, PSMATCH2 etc." accessed 5/28/2020. https://www.statalist.org/forums/forum/general-stata-discussion/general/172008-propensity-score-analysis-for-complex-survey-data-using-pscore-psmatch2-etc

**eTable 1.** Predicted Rates of Each Measure of Quality of End-of-life Care, From Propensity Score Weighted Model

| Supplementary Materials Table 1. Predicted rates of each measure of quality of end-of-life care, from propensity score weighted model. | | |
|---|---|---|
| | **Traditional Medicare** | **Medicare Advantage** |
| Quality of care not rated excellent | 53.1% (50.0%-56.3%) | 59.0% (54.4%-63.5%) |
| Unmet needs pain | 23.9% (19.8%-28.1%) | 27.4% (22.1%-32.7%) |
| Unmet needs dyspnea | 22.8% (17.8%-27.8%) | 25.1% (20.1%-30.2%) |
| Unmet needs anxiety/sadness | 47.3% (42.4%-52.2%) | 53.0% (44.9%-61.1%) |
| Unmet needs spiritual support | 57.2% (53.0%-61.4%) | 57.5% (52.3%-62.8%) |
| Not always treated with respect | 18.5% (15.6%-21.5%) | 19.3% (15.4%-23.2%) |
| Care not consistent with goals | 12.0% (9.2%-14.7%) | 13.9% (10.5%-17.4%) |
| Inadequate Communication | 8.7% (6.8%-10.6%) | 10.7% (7.5%-13.9%) |
| Family not kept informed | 20.9% (18.1%-23.8%) | 27.5% (21.9%-33.2%) |

**eTable 2.** Results of Sensitivity Analysis Defining Medicare Advantage Enrollment at 1 Year Prior to Death

| Supplementary Materials Table 2. Association of MA enrollment one year prior to death with quality of care at the end of life | | |
|---|---|---|
| **Quality Measure** | **OR (95% CI)** | **p-value** |
| Quality of care not rated excellent | 1.21 (0.96-1.53) | 0.10 |
| Unmet needs pain | 1.16 (0.78-1.72) | 0.46 |
| Unmet needs dyspnea | 1.03 (0.65-1.63) | 0.90 |
| Unmet needs anxiety/sadness | 1.30 (0.85-1.99) | 0.22 |
| Unmet needs spiritual support | 1.02 (0.75-1.38) | 0.92 |
| Not always treated with respect | 0.98 (0.69-1.38) | 0.90 |
| Care not consistent with goals | 1.15 (0.77-1.73) | 0.49 |
| Inadequate Communication | 1.28 (0.81-2.05) | 0.29 |
| Family not kept informed | 1.44 (1.06-1.95) | 0.02 |
| Covariates include age at death, female, white race, income quartile, Medicaid enrollment, metropolitan residence, difficulty getting out of bed in last month, ADL impairment at last NHATS survey, probable dementia at last NHATS survey, whether the survey was completed by a child, spouse, or other. All models adjusted by survey design and weights. | | |

© 2020 Ankuda CK et al. *JAMA Network Open.*

**eTable 3.** Comparing Survey Weighted Regression Model and Combined Survey/propensity Weighted Regression Models

| Supplementary Materials Table 3. Predicted rates of each measure of quality of end-of-life care, comparing survey weighted regression model and combined survey/propensity weighted regression models | | | | | | | | | |
|---|---|---|---|---|---|---|---|---|---|
| | **Survey weighted** | | | **Survey weighted regression** | | | **Propensity and survey weighted regression** | | |
| | **Traditional Medicare** | **Medicare Advantage** | **p-value** | **Traditional Medicare** | **Medicare Advantage** | **p-value** | **Traditional Medicare** | **Medicare Advantage** | **p-value** |
| Quality of care not rated excellent | 53.5% (50.9-56.1) | 59.7% (55.4-64.0) | 0.02 | 53.1% (50.0-56.2) | 58.4% (54.0-62.7) | 0.05 | 53.1% (50.0-56.3) | 59.0% (54.4-63.5) | 0.04 |
| Unmet needs pain | 23.1% (19.4-26.8) | 26.1% (20.3-31.9) | 0.77 | 23.9% (19.9-27.9) | 26.8% (21.3-32.3) | 0.45 | 23.9% (19.8-28.1) | 27.4% (22.1-32.7) | 0.36 |
| Unmet needs dyspnea | 20.7% (16.2-25.3) | 23.3% (18.8-27.7) | 0.43 | 22.5% (17.7-27.4) | 22.9% (18.3-27.6) | 0.91 | 22.8% (17.8-27.8) | 25.1% (20.1-30.2) | 0.52 |
| Unmet needs anxiety/sadness | 45.9% (41.9-49.8) | 54.7% (47.1-62.3) | 0.05 | 47.4% (42.6-52.2) | 54.8% (46.3-63.2) | 0.15 | 47.3% (42.4-52.2) | 53.0% (44.9-61.1) | 0.25 |
| Unmet needs spiritual support | 56.6% (52.9-60.3) | 57.5 (52.4-62.7) | 0.78 | 57.7% (53.6-61.8) | 58.4% (52.9-64.0) | 0.84 | 57.2% (53.0-61.4) | 57.5% (52.3-62.8) | 0.93 |
| Not always treated with respect | 17.6% (14.6-20.6) | 21.2% (17.4-25.1) | 0.14 | 18.4% (15.5-21.4) | 18.5% (14.7-22.4) | 0.96 | 18.5% (15.6-21.5) | 19.3% (15.4-23.2) | 0.77 |
| Care not consistent with goals | 11.5% (9.1-14.0) | 13.1% (10.1-16.0) | 0.42 | 12.0% (9.2-14.8) | 13.6% (10.1-17.1) | 0.48 | 12.0% (9.2-14.7) | 13.9% (10.5-17.4) | 0.37 |
| Inadequate Communication | 8.1% (6.5-9.6) | 11.6% (8.3-15.0) | 0.07 | 9.1% (7.1-11.1) | 11.7% (8.2-15.2) | 0.23 | 8.7% (6.8-10.6) | 10.7% (7.5-13.9) | 0.31 |
| Family not kept informed | 20.6% (18.2-23.0) | 26.2% (21.4-31.0) | 0.03 | 21.2% (18.4-24.0) | 27.3% (21.9-32.7) | 0.04 | 20.9% (18.1-23.8) | 27.5% (21.9-33.2) | 0.02 |

© 2020 Ankuda CK et al. *JAMA Network Open.*

**eTable 4.** Full Specifications of the Stratified Analyses Conducted by Hospice Status and Location of Death

| Supplementary Materials Table 4. Full specifications of the stratified analyses by hospice status and location of death. | | | |
|---|---|---|---|
| | Dy/dx | 95% CI | p-value |
| Marginal increase in predicted proportion of those reporting care not excellent, hospice vs. not, additionally adjusting for MA enrollment | -0.128 | -0.202- -0.053 | 0.001 |
| Marginal increase in predicted proportion of those reporting care not excellent, MA vs. not, additionally adjusting for hospice enrollment | 0.683 | 0.171-0.120 | 0.01 |
| Marginal increase in predicted proportion of those reporting care not excellent, MA vs not, within those in NH | 0.207 | 0.081-0.323 | 0.001 |
| Marginal increase in predicted proportion of those reporting care not excellent, MA vs not, within those in hospital | 0.019 | -0.0684-0.107 | 0.663 |
| Marginal increase in predicted proportion of those reporting care not excellent, MA vs not, within those at home | 0.074 | -0.001-0.148 | 0.05 |
| Covariates include age at death, female, white race, income quartile, Medicaid enrollment, metropolitan residence, difficulty getting out of bed in last month, ADL impairment at last NHATS survey, probable dementia at last NHATS survey, whether the survey was completed by a child, spouse, or other. All models adjusted by survey design and weights. | | | |

In stratified analysis for those in both MA and TM, predicted probability of reporting that care is not excellent is higher for those not in hospice compared to those in hospice (Figure 2). However, the predicted probability of reporting that care is not excellent is higher for the family/friends of decedents in MA vs. TM, regardless of hospice enrollment. Stratified by care settings (died at home vs. not, hospital care at end of life vs. not, and nursing home care at end of life vs. not), the gap between MA and TM is greatest for those who received care in the nursing home at the end of life: a predicted probability of 57.2% of family/friends of decedents in TM reported that care was not excellent, compared to 77.9% of family/friends of decedents in MA.

© 2020 Ankuda CK et al. *JAMA Network Open.*
